# Supplementary material for: Body Composition According to Spinal Cord Injury Level: A Systematic Review and Meta-Analysis
Source: J Clin Med. 2021 Aug 30;10(17):3911. doi: 10.3390/jcm10173911 (PMC8432215; doi:10.3390/jcm10173911)
Supplement: Supplementary file 1 [file jcm-10-03911-s001.zip › jcm-1291259-supplementary.pdf]

Online Appendix

Body composition according to the level of spinal cord injury: A systematic review and meta-analysis

Peter Francis Raguindin [1,2,3], Alessandro Bertolo [2]*, Ramona Maria Zeh [2]*, Gion Fränkl [2, 4], Oche Adam Itodo [1,2,3], Simona Capossela [2], Lia Bally [5], Beatrice Minder [6], Mirjam Brach [2], Inge Eriks-Hoogland [2,7], Jivko Stoyanov [2], Taulant Muka [1], Marija Glisic [1,2]

1 Institute of Social and Preventive Medicine (ISPM), University of Bern, Mittelstrasse 43, 3012 Bern, Switzerland
2 Swiss Paraplegic Research, Guido A. Zäch Str. 1, 6207 Nottwil, Switzerland
3 Graduate School for Health Sciences, University of Bern, Mittelsstrasse 43, 3012 Bern Switzerland
4 Graduate School for Cellular and Biomedical Sciences, University of Bern, Mittlestrasse 43, 3012 Bern, Switzerland
5 Department of Diabetes, Endocrinology, Nutritional Medicine and Metabolism, Inselspital, Bern University Hospital and University of Bern, Freiburgstrasse 15, 3010 Bern, Switzerland.
6 Public Health & Primary Care Library, University Library of Bern, University of Bern, Mittelstrasse 43, 3012 Bern, Switzerland
7 Swiss Paraplegic Centre, Guido A. Zäch Strasse 1, 6207 Nottwil, Switzerland
*denotes equal contribution

Corresponding Author
Peter Francis Raguindin
Institute of Social and Preventive Medicine
University of Bern
Mittelstrasse 43, 3012 Bern
peter.raguindin@ispm.unibe.ch

Search strategy

July 22, 2021 (date last searched)

Embase.com

(‘spinal cord injury’/exp OR ‘cervical spine injury’/de OR ‘spinal cord ischemia’/de OR ‘paraplegia’/de OR ‘spastic paraplegia’/de OR ‘quadriplegia’/de OR ‘spinal dysraphism’/de OR (‘injury’/exp AND ‘spinal cord’/exp) OR (((spine or spinal) NEAR/3 (injur* or trauma* or 2ensit*)) OR (‘spinal cord’ NEAR/3 (disease* or contusion* or laceration* or transection* or lesion* or trauma* or ischemi* or ischaemi*)) OR (2ensitive2* NEAR/3 (trauma* or post-trauma* or posttrauma*)) OR ((spine or spinal or vertebra*) NEAR/3 (fracture* or trauma* or injur* or damage* or wound*)) OR ‘central cord injury syndrome*’ OR ‘central cord syndrome*’ OR ‘central spinal cord syndrome*’ OR ‘cauda equine syndrome*’ OR ‘anterior cord syndrome*’ OR ‘conus medullaris syndrome*’ OR ‘Brown Sequard’ OR paraplegi* OR quadriplegi* OR tetraplegi*):ab,ti)

AND

(‘disease severity’/de OR ‘injury severity’/de OR ‘severity of illness index’/exp OR ‘disease marker’/de OR ‘American Spinal Injury Association impairment scale’/de OR (((level* OR severit* OR completeness OR degree* OR marker*) NEAR/6 (injur* OR lesion* OR illness* OR disease* OR SCI)) OR (neurologic* NEAR/1 level*) OR (lesion* NEAR/3 dependen*)):ab,ti)

AND

(‘diabetes mellitus’/exp OR ‘cardiovascular disease’/de OR ‘heart failure’/de OR ‘congestive heart failure’/de OR ‘heart disease’/de OR ‘cardiovascular risk’/exp OR ‘cardiometabolic risk’/de OR ‘cardiovascular mortality’/de OR ‘blood pressure’/exp OR ‘hypertension’/de OR ‘coronary artery disease’/de OR ‘ischemic heart disease’/exp OR ‘cerebrovascular accident’/de OR ‘venous thromboembolism’/de OR ‘atherosclerotic cardiovascular disease’/de OR ‘brain ischemia’/exp OR ‘insulin response’/exp OR ‘glucose blood level’/exp OR ‘insulin blood level’/exp OR ‘hyperinsulinism’/exp OR ‘lipid blood level’/exp OR ‘lipid level’/exp OR ‘glycosylated hemoglobin’/exp OR ‘C reactive protein’/de OR ‘c reactive protein blood level’/de OR (inflammation/de AND (marker/de OR ‘C reactive protein’/exp OR cytokine/de OR fibrinolysis/exp OR ‘tumor necrosis factor alpha’/exp)) OR ‘chronic inflammation’/exp OR atherosclerosis/de OR ‘atherosclerotic plaque’/de OR ‘carotid atherosclerosis’/exp OR ‘coronary artery atherosclerosis’/exp OR ‘obesity’/de OR ‘body mass’/de OR ‘abdominal obesity’/de OR ‘waist circumference’/de OR ‘intra-abdominal fat’/exp OR ‘body composition’/exp OR ‘metabolic disorder’/exp OR ‘oxidative stress’/de OR ‘reactive oxygen metabolite’/de OR ‘lipid peroxidation’/de OR ‘isoprostane derivative’/de OR ‘malonaldehyde’/de OR ‘lipoxygenase’/de OR ‘myeloperoxidase’/de OR ‘endothelium derived relaxing factor’/de OR ‘nitric oxide’/de OR ‘prostacyclin derivative’/exp OR ‘endothelium derived hyperpolarizing factor’/de OR ‘endothelium derived constricting factor’/de OR ‘endothelin 1’/de OR ‘intercellular adhesion molecule 1’/de OR ‘vascular cell adhesion molecule 1’/de OR ‘endothelial leukocyte adhesion molecule 1’/de OR ‘PADGEM protein’/de OR ‘arterial stiffness’/de OR ‘heart function’/exp OR ‘cardiovascular function’/exp OR ‘flow-mediated dilation test’/de OR ‘peripheral arterial tonometry’/de OR ‘heart stroke volume’/de OR (diabet* OR HbA1c OR HbA-1c OR ((cardiovascular OR coronar* OR cardiac OR heart OR cardiometabol* OR cardio-metabol* OR metabolic) NEAR/3 (disease* OR event* OR disorder* OR syndrome* OR function* OR dysfunction* OR health OR mortality OR risk*)) OR cvd OR cvds OR CV-risk OR ‘blood pressure*’ OR hypertension OR ((ischemi* OR ischaemi* OR fail* OR insufficien* OR infarct*) NEAR/3 (heart OR cardia* OR myocard*)) OR (cerebrovascular* NEAR/3 accident*) OR cva OR stroke* OR cardiopath* OR angina OR ((brain OR cerebral) NEAR/3 (ischemi* OR ischaemi*)) OR ((glucose OR sugar OR insulin* OR lipid* OR cholester* OR lipoprotein* OR triacylglycerol* OR triglyceride*) NEAR/6 (level* OR blood OR serum OR plasma* OR concentration*)) OR dyslipidemia* OR dyslipidaemia* OR glucosaem* OR 2ensiti* OR glycaem* OR glycem* OR hyperinsulin* OR hypoinsulin* OR insulinaem* OR insulinem* OR (insulin NEAR/3 (response OR dependen* OR resistan* OR 2ensitive*)) OR hypercholesterol* OR (inflammat* NEAR/3 (chronic* OR marker* OR biomarker* OR interleukin* OR crp OR ‘c reactive’ OR cytokine* OR leptin* OR fibrinolys* OR fibrinogenlys* OR ‘tumor necrosis factor’ OR tnf)) OR atheroscler* OR arterioscler* OR homocysteine* OR obes* OR adipos* OR (waist NEAR/3 (circumference* or hip)) OR ((body) NEAR/3 (mass OR size OR weight OR composition* OR fat OR lipid*)) OR ‘muscle loss’ OR bmi OR (oxidative NEAR/3 stress*) OR (reactive NEAR/3 oxygen* NEAR/3 (metabolite* OR species)) OR (lipid* NEAR/3 (peroxidat* OR autooxidat* OR autoxidat*)) OR lipoperoxidat* OR lipo-peroxidat* OR isoprostan* OR malonaldehyde* OR lipoxygenase* OR myeloperoxidase* OR ‘endothelial-derived relaxing factor’ OR ‘nitric oxide’ OR prostaglandin-i2 OR PGI2 OR ‘endothelium derived 2ensitive22zing factor*’ OR ‘endothelium derived constricting factor*’ OR ‘endothelium derived contracting factor*’ OR endothelin-1 OR ‘intercellular adhesion molecule-1’ OR ‘vascular cell adhesion molecule-1’ OR E-selectin OR selectin-E OR P-selectin OR selectin-P OR ((vascular OR arterial) NEAR/1 (stiffness)) OR ((arterial) NEAR/1 (wall OR thickness)) OR ((ventricular OR ventricle OR heart) NEAR/3 (function* OR

dysfunction* OR rate*)) OR 'ventricle stroke volume' OR 'pulse wave' OR 'flow-mediated dilatation' OR 'peripheral arterial tonometry' OR echocardiograph*):ab,ti)

NOT ([animals]/lim NOT [humans]/lim) NOT ([Conference Abstract]/lim OR [Letter]/lim OR [Note]/lim OR [Editorial]/lim)

Medline Ovid

(exp Spinal Cord Injuries/ OR exp Spinal Cord Ischemia/ OR exp Paraplegia/ OR Quadriplegia/ OR Spinal Dysraphism/ OR (((spine or spinal) ADJ3 (injur* or trauma* or 3ensit*)) OR (spinal cord ADJ3 (disease* or contusion* or laceration* or transection* or lesion* or trauma* or ischemi* or ischaemi*)) OR (3ensitive3* ADJ3 (trauma* or post-trauma* or posttrauma*)) OR ((spine or spinal or vertebra*) ADJ3 (fracture* or trauma* or injur* or damage* or wound*)) OR central cord injury syndrome* OR central cord syndrome* OR central spinal cord syndrome* OR cauda equine syndrome* OR anterior cord syndrome* OR conus medullaris syndrome* OR Brown Sequard OR paraplegi* or quadriplegi* or tetraplegi*).ab,ti.)

AND

("Severity of Illness Index"/ OR (((level* OR severit* OR completeness OR degree* OR marker*) ADJ6 (injur* OR lesion* OR illness* OR disease* OR SCI)) OR (neurologic* ADJ1 level*) OR (lesion* ADJ3 dependen*)).ab,ti.)

AND

(exp "Diabetes Mellitus"/ OR "Cardiovascular Diseases"/ OR "Heart Failure"/ OR "Heart Diseases"/ OR exp Blood Pressure/ OR exp "Coronary Artery Disease"/ OR exp "Myocardial Ischemia"/ OR exp "Stroke"/ OR Venous Thromboembolism/ OR Hypertension/ OR "Atherosclerosis"/ OR exp "Brain Ischemia"/ OR "Insulin Resistance"/ OR glucose/bl OR insulin/bl OR exp Hyperinsulinism/ OR lipids/bl OR Glycated Hemoglobin A/ OR C-Reactive Protein/OR (inflammation/ AND (biomarkers/ OR "C-Reactive Protein"/ OR cytokines/ OR fibrinolysis/ OR "Tumor Necrosis Factor-alpha"/)) OR Atherosclerosis/ OR "Plaque, Atherosclerotic"/ OR "Carotid Artery Diseases"/ OR exp obesity/ OR "Body Mass Index"/ OR Waist Circumference/ OR Intra-Abdominal Fat/ OR exp Body Composition/ OR Metabolic Diseases/ OR Oxidative Stress/ OR Reactive Oxygen Species/ OR Lipid Peroxidation/ OR Isoprostanes derivative/ OR Malondialdehyde/ OR Lipoxygenase/ OR Peroxidase/ OR Endothelium-Dependent Relaxing Factors/ OR Nitric Oxide/ OR exp prostaglandins i/ OR Endothelin-1/ OR Nitrites/ OR Cell Adhesion Molecules/ OR Intercellular Adhesion Molecule-1/ OR Vascular Cell Adhesion Molecule-1/ OR e-selectin/ OR p-selectin/ OR Vascular Stiffness/ OR Pulse Wave Analysis/ OR exp Heart Function Tests/ OR Ventricular Dysfunction/ OR (diabet* OR HbA1c OR HbA-1c OR ((cardiovascular OR coronar* OR cardiac OR heart OR cardiometabol* OR cardio-metabol* OR metabolic) ADJ3 (disease* OR event* OR disorder* OR syndrome* OR function* OR dysfunction* OR health OR mortality OR risk*)) OR cvd OR cvds OR CV-risk OR blood pressure* OR ((ischemi* OR ischaemi* OR fail* OR insufficien* OR infarct*) ADJ3 (heart OR cardia* OR myocard*)) OR (cerebrovascular* ADJ3 accident*) OR cva OR stroke* OR cardiopath* OR angina OR ((brain OR cerebral) ADJ3 (ischemi* OR ischaemi*)) OR ((glucose OR sugar OR insulin* OR lipid* OR cholester* OR lipoprotein* OR triacylglycerol* OR triglyceride*) ADJ6 (level* OR blood OR serum OR plasma* OR concentration*)) OR dyslipidemia* OR dyslipidaemia* OR glucosaem* OR 3ensiti* OR glycaem* OR glycem* OR hyperinsulin* OR hypoinsulin* OR insulinaem* OR insulinem* OR (insulin ADJ3 (response OR dependen* OR resistan* OR 3ensitive*)) OR hypercholesterol* OR (inflammat* ADJ3 (chronic* OR marker* OR biomarker* OR interleukin* OR crp OR c reactive OR cytokine* OR leptin* OR fibrinolys* OR fibrinogenlys* OR tumor necrosis factor OR tnf)) OR atheroscler* OR arterioscler* OR homocysteine* OR obes* OR adipos* OR (waist ADJ3 (circumference* or hip)) OR ((body) ADJ3 (mass OR size OR weight OR composition* OR fat OR lipid*)) OR muscle loss OR bmi OR (oxidative ADJ3 stress*) OR (reactive ADJ3 oxygen* ADJ3 (metabolite* OR species)) OR (lipid* ADJ3 (peroxidat* OR autooxidat* OR autoxidat*)) OR lipoperoxidat* OR lipo-peroxidat* OR isoprostan* OR malonaldehyde* OR lipoxygenase* OR myeloperoxidase* OR endothelial-derived relaxing factor OR nitric oxide OR prostaglandin-i2 OR PGI2 OR endothelium derived 3ensitive33zing factor* OR endothelium derived constricting factor* OR endothelium derived contracting factor* OR endothelin-1 OR intercellular adhesion molecule-1 OR vascular cell adhesion molecule-1 OR E-selectin OR selectin-E OR P-selectin OR selectin-P OR ((vascular OR arterial) ADJ1 (stiffness)) OR ((arterial) ADJ1 (wall OR thickness)) OR ((ventricular OR ventricle OR heart) ADJ3 (function OR dysfunction OR rate)) OR ventricle stroke volume OR pulse wave OR flow-mediated dilatation OR peripheral arterial tonometry OR echocardiograph*).ab,ti.)

NOT (exp animals/ NOT humans/) NOT (letter OR news OR comment OR editorial OR congress).pt.

Cochrane CENTRAL: Cochrane Central Register of Controlled Trials, Issue 7 of 12, July 2020

((((spine or spinal) NEAR/3 (injur* or trauma* or 4ensit*)) OR ("spinal cord" NEAR/3 (disease* or contusion* or laceration* or transection* or lesion* or trauma* or ischemi* or ischaemi*)) OR (4ensitive4* NEAR/3 (trauma* or post-trauma* or posttrauma*)) OR ((spine or spinal or vertebra*) NEAR/3 (fracture* or trauma* or injur* or damage* or wound*)) OR "central cord injury syndrome" OR "central cord syndrome" OR "central spinal cord syndrome" OR "cauda equine syndrome" OR "anterior cord syndrome" OR "conus medullaris syndrome" OR "Brown Sequard" OR paraplegi* OR quadriplegi* OR tetraplegi*)):ab,ti,kw

AND

((((level* OR severit* OR completeness OR degree* OR marker*) NEAR/6 (injur* OR lesion* OR illness* OR disease* OR SCI)) OR (neurologic* NEAR/1 level*) OR (lesion* NEAR/3 dependen*))):ab,ti,kw

AND

((diabet* OR HbA1c OR HbA-1c OR ((cardiovascular OR coronar* OR cardiac OR heart OR cardiometabol* OR cardio-metabol* OR metabolic) NEAR/3 (disease* OR event* OR disorder* OR syndrome* OR function* OR dysfunction* OR health OR mortality OR risk*)) OR cvd OR cvds OR CV-risk OR blood-pressure* OR hypertension OR ((ischemi* OR ischaemi* OR fail* OR insufficien* OR infarct*) NEAR/3 (heart OR cardia* OR myocard*)) OR (cerebrovascular* NEAR/3 accident*) OR cva OR stroke* OR cardiopath* OR angina OR ((brain OR cerebral) NEAR/3 (ischemi* OR ischaemi*)) OR ((glucose OR sugar OR insulin* OR lipid* OR cholester* OR lipoprotein* OR triacylglycerol* OR triglyceride*) NEAR/6 (level* OR blood OR serum OR plasma* OR concentration*)) OR dyslipidemia* OR dyslipidaemia* OR glucosaem* OR 4ensiti* OR glycaem* OR glycem* OR hyperinsulin* OR hypoinsulin* OR insulinaem* OR insulinem* OR (insulin NEAR/3 (response OR dependen* OR resistan* OR 4ensitive*)) OR hypercholesterol* OR (inflammat* NEAR/3 (chronic* OR marker* OR biomarker* OR interleukin* OR crp OR c-reactive OR cytokine* OR leptin* OR fibrinolys* OR fibrinogenlys* OR "tumor necrosis factor" OR tnf)) OR atheroscler* OR arterioscler* OR homocysteine* OR obes* OR adipos* OR (waist NEAR/3 (circumference* or hip)) OR ((body) NEAR/3 (mass OR size OR weight OR composition* OR fat OR lipid*)) OR "muscle loss" OR bmi OR (oxidative NEAR/3 stress*) OR (reactive NEAR/3 oxygen* NEAR/3 (metabolite* OR species)) OR (lipid* NEAR/3 (peroxidat* OR autooxidat* OR autoxidat*)) OR lipoperoxidat* OR lipo-peroxidat* OR isoprostan* OR malonaldehyde* OR lipoxygenase* OR myeloperoxidase* OR "endothelial-derived relaxing factor" OR "nitric oxide" OR prostaglandin-i2 OR PGI2 OR endothelium-derived-hyperpolarising-factor* OR endothelium-derived-constricting-factor* OR endothelium-derived-contracting-factor* OR endothelin-1 OR "intercellular adhesion molecule-1" OR "vascular cell adhesion molecule-1" OR E-selectin OR selectin-E OR P-selectin OR selectin-P OR ((vascular OR arterial) NEAR/1 (stiffness)) OR ((arterial) NEAR/1 (wall OR thickness)) OR ((ventricular OR ventricle OR heart) NEAR/3 (function* OR dysfunction* OR rate*)) OR "ventricle stroke volume" OR "pulse wave" OR "flow-mediated dilatation" OR "peripheral arterial tonometry" OR echocardiograph*)):ab,ti,kw

Web of Science

TS=((((spine or spinal) NEAR/3 (injur* or trauma* or 4ensit*)) OR ("spinal cord" NEAR/3 (disease* or contusion* or laceration* or transection* or lesion* or trauma* or ischemi* or ischaemi*)) OR (4ensitive4* NEAR/3 (trauma* or post-trauma* or posttrauma*)) OR ((spine or spinal or vertebra*) NEAR/3 (fracture* or trauma* or injur* or damage* or wound*)) OR "central cord injury syndrome" OR "central cord syndrome" OR "central spinal cord syndrome" OR "cauda equine syndrome" OR "anterior cord syndrome" OR "conus medullaris syndrome" OR "Brown Sequard" OR paraplegi* OR quadriplegi* OR tetraplegi*))

AND

TS=((((level* OR severit* OR completeness OR degree* OR marker*) NEAR/6 (injur* OR lesion* OR illness* OR disease* OR SCI)) OR (neurologic* NEAR/1 level*) OR (lesion* NEAR/3 dependen*)))

AND

TS=((diabet* OR HbA1c OR HbA-1c OR ((cardiovascular OR coronar* OR cardiac OR heart OR cardiometabol* OR cardio-metabol* OR metabolic) NEAR/3 (disease* OR event* OR disorder* OR syndrome* OR function* OR dysfunction* OR health

OR mortality OR risk*)) OR cvd OR cvds OR CV-risk OR “blood pressure*” OR hypertension OR ((ischemi* OR ischaemi* OR fail* OR insufficien* OR infarct*) NEAR/3 (heart OR cardia* OR myocard*)) OR (cerebrovascular* NEAR/3 accident*) OR cva OR stroke* OR cardiopath* OR angina OR ((brain OR cerebral) NEAR/3 (ischemi* OR ischaemi*)) OR ((glucose OR sugar OR insulin* OR lipid* OR cholester* OR lipoprotein* OR triacylglycerol* OR triglyceride*) NEAR/6 (level* OR blood OR serum OR plasma* OR concentration*)) OR dyslipidemia* OR dyslipidaemia* OR glucosaem* OR 5ensiti* OR glycaem* OR glycem* OR hyperinsulin* OR hypoinsulin* OR insulinaem* OR insulinem* OR (insulin NEAR/3 (response OR dependen* OR resistan* OR 5ensitive*)) OR hypercholesterol* OR (inflammat* NEAR/3 (chronic* OR marker* OR biomarker* OR interleukin* OR crp OR c-reactive OR cytokine* OR leptin* OR fibrinolys* OR fibrinogenlys* OR “tumor necrosis factor*” OR tnf)) OR atheroscler* OR arterioscler* OR homocysteine* OR obes* OR adipos* OR (waist NEAR/3 (circumference* or hip)) OR ((body) NEAR/3 (mass OR size OR weight OR composition* OR fat OR lipid*)) OR “muscle loss” OR bmi OR (oxidative NEAR/3 stress*) OR (reactive NEAR/3 oxygen* NEAR/3 (metabolite* OR species)) OR (lipid* NEAR/3 (peroxidat* OR autooxidat* OR autoxidat*)) OR lipoperoxidat* OR lipo-peroxidat* OR isoprostan* OR malonaldehyde* OR lipoxygenase* OR myeloperoxidase* OR “endothelial-derived relaxing factor” OR “nitric oxide” OR prostaglandin-i2 OR PGI2 OR “endothelium-derived hyperpolarizing factor*” OR “endothelium-derived constricting factor*” OR “endothelium-derived contracting factor*” OR endothelin-1 OR “intercellular adhesion molecule-1” OR “vascular cell adhesion molecule-1” OR E-selectin OR selectin-E OR P-selectin OR selectin-P OR ((vascular OR arterial) NEAR/1 (stiffness)) OR ((arterial) NEAR/1 (wall OR thickness)) OR ((ventricular OR ventricle OR heart) NEAR/3 (function* OR dysfunction* OR rate*)) OR “ventricle stroke volume*” OR “pulse wave*” OR “flow-mediated dilatation*” OR “peripheral arterial tonometry” OR echocardiograph*))

NOT TS=(((animal* OR rat OR rats OR mouse OR mice OR murine OR nonhuman* OR primate*) NOT (human* OR patient*)))

AND DT=(article)

Google scholar

16400 results (22.07.2021) first 400 according to relevance ranking

Spinal Cord|SCI “level|levels of injury”|”lesion level”|”level of lesions”|”injury level|levels”
diabetes|”cardiovascular|coronary|metabolic|cardiometabolic
disease|events|syndromes|risk|mortality”|stroke|obesity|”body mass|composition|fat|weight” -mice

PRISMA 2009 Checklist

Section/topic	#	Checklist item	Reported on page #
**TITLE**			
Title	1	Identify the report as a systematic review, meta-analysis, or both.	P1 L2-3
**ABSTRACT**			
Structured summary	2	Provide a structured summary including, as applicable: background; objectives; data sources; study eligibility criteria, participants, and interventions; study appraisal and synthesis methods; results; limitations; conclusions and implications of key findings; systematic review registration number.	P1 L17-31
**INTRODUCTION**			
Rationale	3	Describe the rationale for the review in the context of what is already known.	P2 L50-58
Objectives	4	Provide an explicit statement of questions being addressed with reference to participants, interventions, comparisons, outcomes, and study design (PICOS).	P2 L59-64
**METHODS**			
Protocol and registration	5	Indicate if a review protocol exists, if and where it can be accessed (e.g., Web address), and, if available, provide registration information including registration number.	P2 L68-69
Eligibility criteria	6	Specify study characteristics (e.g., PICOS, length of follow-up) and report characteristics (e.g., years considered, language, publication status) used as criteria for eligibility, giving rationale.	P2 S2.1.
Information sources	7	Describe all information sources (e.g., databases with dates of coverage, contact with study authors to identify additional studies) in the search and date last searched.	P2 L73-75
Search	8	Present full electronic search strategy for at least one database, including any limits used, such that it could be repeated.	Supplement
Study selection	9	State the process for selecting studies (i.e., screening, eligibility, included in systematic review, and, if applicable, included in the meta-analysis).	P2 L85-91
Data collection process	10	Describe method of data extraction from reports (e.g., piloted forms, independently, in duplicate) and any processes for obtaining and confirming data from investigators.	P2-3 S2.3-2.4
Data items	11	List and define all variables for which data were sought (e.g., PICOS, funding sources) and any assumptions and simplifications made.	P2 L 77-81
Risk of bias in individual studies	12	Describe methods used for assessing risk of bias of individual studies (including specification of whether this was done at the study or outcome level), and how this information is to be used in any data synthesis.	P3 S2.4.
Summary measures	13	State the principal summary measures (e.g., risk ratio, difference in means).	P3 S2.5

Synthesis of results	14	Describe the methods of handling data and combining results of studies, if done, including measures of consistency (e.g., I^2) for each meta-analysis.	P3-4 S2.5
Risk of bias across studies	15	Specify any assessment of risk of bias that may affect the cumulative evidence (e.g., publication bias, selective reporting within studies).	P3-4 S2.5
Additional analyses	16	Describe methods of additional analyses (e.g., sensitivity or subgroup analyses, meta-regression), if done, indicating which were pre-specified.	P3-4 S2.5
**RESULTS**			
Study selection	17	Give numbers of studies screened, assessed for eligibility, and included in the review, with reasons for exclusions at each stage, ideally with a flow diagram.	P4 L167-174
Study characteristics	18	For each study, present characteristics for which data were extracted (e.g., study size, PICOS, follow-up period) and provide the citations.	Supplement
Risk of bias within studies	19	Present data on risk of bias of each study and, if available, any outcome level assessment (see item 12).	Supplement
Results of individual studies	20	For all outcomes considered (benefits or harms), present, for each study: (a) simple summary data for each intervention group (b) effect estimates and confidence intervals, ideally with a forest plot.	Supplement
Synthesis of results	21	Present results of each meta-analysis done, including confidence intervals and measures of consistency.	Table 2<br>S3.2-S3.4
Risk of bias across studies	22	Present results of any assessment of risk of bias across studies (see Item 15).	Supplement<br>P4 L190-192
Additional analysis	23	Give results of additional analyses, if done (e.g., sensitivity or subgroup analyses, meta-regression [see Item 16]).	Supplement<br>P9 S3.5-S3.7
**DISCUSSION**			
Summary of evidence	24	Summarize the main findings including the strength of evidence for each main outcome; consider their relevance to key groups (e.g., healthcare providers, users, and policy makers).	P9 L314-319
Limitations	25	Discuss limitations at study and outcome level (e.g., risk of bias), and at review-level (e.g., incomplete retrieval of identified research, reporting bias).	P11-12 S4.3
Conclusions	26	Provide a general interpretation of the results in the context of other evidence, and implications for future research.	P12 L441-449
**FUNDING**			
Funding	27	Describe sources of funding for the systematic review and other support (e.g., supply of data); role of funders for the systematic review.	P13 L462-465

From: Moher D, Liberati A, Tetzlaff J, Altman DG, The PRISMA Group (2009). Preferred Reporting Items for Systematic Reviews and Meta-Analyses: The PRISMA Statement. PLoS Med 6(7): e1000097. doi:10.1371/journal.pmed1000097

Table S1. Summary of studies included in the meta-analysis

Author, year of publication	Study Design	Study Location (country)	Population (setting)	No. of individuals	Sex, number (% male)	SCI lesion duration, years (mean ±SD)	Age, years (mean ±SD)	Classification of injury	Complete vs incomplete	Outcomes (Body composition focus, Y)	CV disease and meds	Study Quality
Akbal 2013[1]	CS	Turkey	Ankara Rehabilitation Research hospital, Jan 2008 to Aug 2009, (no cardiovascular disease baseline)	56	n.a.	3.73 (7.66)	32.46 (14.2)	TP vs PP	13%	BMI, waist circ (No)	No CAD	9
Azevedo 2016[2]	CS	US	University Hospital of UNICAMP (injury of more than 2 years)	39	39 (100)	*>2 y	36.1 (8.6)	TP vs PP	5%	BMI, fat mass, lean mass (Yes)	n.d.	8
Baumann 1999 [3]	CS	US	Los Amigos Medical Center, May 1994-May 1998	201	169 (84.1)	13 (0.7)	39 (0.8)	TP vs PP	100%	BMI, fat % (Yes)	n.d.	8
Baumann 1992 [4]	CS	US	Veterans Affairs Medical Center	100	100 (100)	16.3 (1.7)	47.9 (1.95)	TP vs PP	n.d.	BMI (No)	n.d.	7
Buchholz 2009 [5]	CS	Canada	Multicenter in Ontario, 2008-2010, (has cross sectional and cohort parts)	56	44 (78.5)	14.55 (10.1)	41.85 (12.22)	TP vs PP	n.d.	BMI, fat %, waist circ (Yes)	Included CVD and CV meds but not specified	9
Chun 2017 [6]	CS	South Korea	Seoul National Rehabilitation Hospital, South Korea	50	38 (76)	12.2 (7.4)	41.9 (10.7)	TP vs PP	100%	BMI, fat % (Yes)	n.d.	8
de Groot [7]	CH	Netherlands	8 Dutch rehabilitation center, ALLRISC consortium,	115	84 (73)	22.7 (5.0)	41.4 (14.05)	TP vs PP	53%	BMI (No)	n.d.	9
Dionyssiotis 2008 [8]	CS	Greece	National Rehabilitation Center in Athens, Greece	31	31 (100)	5.8 (5.9)	39.2 (15)	PP-H <T7<br>PP-L >T7	100%	BMI, fat % (Yes)	n.d.	8
Farkas 2019 [9]	CS	US	Spinal cord injury registry, Pennsylvania, US	41	35 (85)	14.8 (11.4)	44.2 (11.1)	TP vs PP	100%	Fat % (Yes)	Excluded CVD, DM and smokers	8
Flueck 2020[10]	CS	Switzerland	Swiss Paraplegic Center, Switzerland (Paralympic athletes)	69	49 (71.0)	19 (11)	33 (11)	TP vs PP	n.a.	Weight, VAT/SAT	n.d.	6
Gater 2021 [11]	CS	US	Virginia Commonwealth University (VCU) General Clinical Research Center (GCRC) and Hunter Holmes McGuire VAMC (HHMVAMC)	72	59 (82)	14.4 (11.1)	44.4 (11.3)	TP vs PP	100%	BMI, waist circumference	n.d.	8
Gibson 2008 [12]	CS	Canada	Community-based cohort in Ontario, SHAPE-SCI consortium	69	56 (81.1)	14.7 (10.4)	42.4 (12.0)	TP vs PP	38%	BMI, fat %, waist circ (Yes)	n.d	9
Gorgey 2011A [13]	CS	US	Veterans Affairs Medical Center, Virginia, US	32	32 (100)	n.d.	36 (9)	TP vs PP	100%	BMI, lean mass, fat mass, fat % (Yes)	CVD and DM excluded	8
Gomes Costa [14]	CS	Brazil	Rede Sarah de Hospitais de Rehabiliticao, Brasilia	45	45 (100)	2.3 (2.8)	27.6 (6.6)	TP C6-C8<br>PP-H T1-T6<br>PP-L T7-12	100%	BMI (no)	Excluded CVD and diabetes	7
Han 2015 [15]	CS	South Korea	National Rehabilitation Center, Seoul, South Korea	915	640 (69.9) with sex analysis	6.0 (6.4)	45.2 (14.6)	TP vs PP	n.d.	BMI, fat % (Yes)	n.d.	8
Inukai 2006 [16]	CS	Japan	Okayama prefecture	25	25 (100)	15.7 (8.8)	35.6 (9.7)	PP-H T7 up<br>PP-L T7 down	n.d.	BMI, fat %, waist circ (Yes)	n.d.	6
Janssen 1997 [17]	CS	Netherlands	Vriej Universitet, Amsterdam, Netherlands	37	37 (100)	14.7 (8.6)	37.4 (12.0)	TP C4-C8<br>PP-H T1-T5<br>PP-M T6-T10<br>PP-L T11-L5	62%	BMI (No)	No DM, 1 CAD, no CVD meds	8

Katzelnick 2017 [18]	CS	US	Kessler foundation, NJ, and Veterans Affairs Medical Center, NY	46	38 (82.6)	10.6 (6.96)	35.3 (7.0)	H C3-T5<br>L T7-12	45.6%	BMI (No)	n.d.	8
Katzelnick 2019 [19]	CS	US	Kessler foundation, NJ, and Veterans Affairs Medical Center, NY	113	97 (85.8)	15.7 (13.4)	51.5 (15)	TP C1-T1<br>PP-H T2-4<br>PP-L T5-12	40.7%	BMI (No)	n.d.	8
Kemp 2000 [20]	CS	US	Rancho Los Amigos National Rehabilitation Center in California	188	152 (81)	16 (9)	42 (11)	TP vs PP	68%	BMI, fat % (Yes)	n.d.	8
Kim 2016 [21]	CS	US	Elite paracyclists participating in 2014 United Cycliste Internationale Para-Cycling Road World Cycling Championships	44	30 (68)	n.d.	39.8 (8.7)	TP C1-C8<br>PP T1-5<br>PP >T6	n.d.	BMI (No)	No CVD	8
Lee 2017 [22]	CC	South Korea	Wonju Severance Christian Hospital, South Korea	15	13 (87)	12.33 (5.99)	37.78 (11.23)	H <T6<br>L >T7	n.d.	BMI (No)	Excluded CV disease and meds	8
Li 2019 [23]	CS	US	University of Alabama at Birmingham, Alabama, US	22	0 (0)	13.9 (13.2)	42.4 (10.9)	TP vs PP	n.d.	BMI, fat %, fat mass, lean mass	Excluded CV diseases and med	8
Matos-Souza 2010 [24]	CS	Brazil	Rehabilitation center in Sao Paulo, Brazil	34	34 (100)	6.7 (1.2)	31.9 (1.8)	TP vs PP	n.d.	BMI (No)	Excluded CV disease and meds	8
McCaulay 2018 [25]	CS	US	McGuire VA Medical Center	22*	22 (100)	8.3 (7.8)	37 (10.3)	TP vs PP	n.d.	BMI, waist circ (Yes)	Excluded CV disease and meds	8
Miyatami 2014 [26]	CS	Canada	Toronto Rehabilitation Institute, Toronto, Canada	87	65 (74.7)	13.5 (4.6)	48.1 (13.7)	TP C28<br>PP T1-12	n.d.	BMI, waist circ (No*)	Excluded CV disease and meds	9
O Brien 2017 [27]	CS	US	McGuire VA Medical Center, Virginia, US	22	22 (100)	8.2 (7.9)	36.1 (10.0)	TP vs PP	72%	BMI (No)	Excluded CV disease and meds	8
Pelletier 2016 [28]	CS	Canada	Lyndhurst Center, Toronto Rehabilitation Institute	136	100 (73.5)	15.6 (11.3)	49.1 (12.9)	TP vs PP	63.2%	BMI, waist circ, fat %	n.d.	8
Powell 2017 [29]	CH	US	National Spinal Cord Injury Database	1094	881 (80.6) has sex analysis	n.d.	40.9 (16.9)	TP vs PP	n.d.	BMI (No)	n.d.	8
Rankin 2017 [30]	CS	US	McGuire VA Medical Center	22	22 (100)	8.2 (7.9)	36.1 (10.0)	TP vs PP	100%	BMI, VAT	Excluded those with CV disease and diabetes	7
Raymond 2010 [31]	CS	Australia	New South Wales, Australia (excluded all patients with cardiovascular morbidity)	25	20 (80)	11.0 (7.6)	37 (9)	TP C2-8<br>PP-H T1-5<br>PP-L T6-12	28%	BMI (No)	Excluded CV disease and meds	8
Ribiero Neto 2013 [32]	CS	Brazil	Centro Internacional de Neurociências e Reabilitação Sarah, Brasília,	74	74 (100)	3.5 (2.5)	27.1 (7.2)	TP C4-C8<br>PP-H T1-T6<br>PP-L T7-L3	66.2%	BMI, fat % (Yes)	n.d.	8
Sabour 2013 [33]	CS	Iran	Brain and Spinal Injury Research Center at Tehran University of Medical Sciences, Iran, May 2008 - June 2009	162	131 (80.9)	8.03 (5.75)	34.17 (8.75)	TP vs PP	29.6%	BMI, waist circ (No*)	Excluded DM	8
Spungen 2003 [34]	CS	US	Rancho Los Amigos National Rehabilitation Center, California	133	133 (100)	13.0 (1.1)	38.5 (1.4)	TP vs PP	70.6%	BMI, fat mas, lean mass, fat % (Yes)	n.d.	8
Sumrell 2018[35]	CS	US	Hunter Holmes McGuire VA Center and Virginia Commonwealth University Richmond	22	22 (100)	8 (8)	36 (10)	TP vs PP	100%	BMI, VAT/SAT	Excluded CVD and DM	7
Wang 2005 [36]	CS	Taiwan	National Taiwan University Hospital	110	110 (100)	10.8 (0.7)	39.6 (10.3)	SCI-H Above T6,	100% complete	BMI (No)	n.d.	9

								SCI-L Below T6				
Wang 2007 [37]	CS	Taiwan	University Hospital in Taiwan	89	89 (100)	10.8 (0.7)	39.3 (1.1)	TP T1 above<br>PP Below T1	100% complete	BMI (No)	n.d.	8
Yahiro 2019 [38]	CS	US	Edward Hines, Jr. Veterans Affairs (VA) Hospital in Hines, Illinois, US	155	155 (100)	18.7 (14.3)	61.1 (13.7)	TP vs PP	29.0%	BMI, waist circ (N0)	62% taking meds for DM, HTN and/or hyperlipidemia	8
Yilmaz 2007 [39]	CS	Turkey	Armed Forces Rehabilitation Center, Ankara, Turkey	30	30 (100)	2.5 y	32 (10)	TP vs PP	100%	Fat mass, lean mass	Excluded with cardiovascular diseases	7
Zhu 2013 [40]	CS	US	Veterans Affairs Medical Center, New York, USA	277	271 (98)	18 (15)	63 (14)	TP C1-8<br>PP-H T1-6<br>PP-L T7-12	n.d.	BMI (No)	Included CV disease	9

*n.d., no data; TP, PP

*focus on body composition but results cannot be pooled

Table S2. Characteristics of studies that were excluded for meta-analysis

Author-Year	Study design (Location)	Participants (% Male)	Age, mean y (±SD)	SCI lesion duration (mean y±SD)	Outcomes	Results comparing body morphology	Reason for non-abstraction of data
Astorino 2015 [41]	CH (US)	17 (88.2)	36.1 (11.5)	2.1 (2.9)	BMI, fat %, fat mass	No difference was observed between paraplegia and tetraplegia (p value 0.20)	No disaggregated data reported
Baumann 1994 [42]	CS (US)	100 (100)	49 (2)	18.3 (2)	BMI	There were no significant differences between tetraplegics and paraplegics on body mass index	No disaggregated data for tetraplegics and paraplegics
Bernardi 2019 [43]	CS (Italy)	25 (100%)	38.42 (12.22)	n.a.	BMI, waist circumference	Waist circumference is higher in Paraplegia-High (82 cm; range 76-98) compared to Paraplegia-Low (80 cm; range 77-90).	No other studies reported waist circumference for other level of paraplegia
Brenes 1986 [44]	CS (US)	56 (84.4)	34.8 (6.8)	*53% >1 y	BMI	There were no significant differences between controls and SCI group	No reported exact value comparing body morphology on different injury levels.
Dionyssiotis 2009 [45]	CC (Greece)	30 (100)	39 (15.0)	n.a.	BMI, lean mass, fat mass	Paraplegics have significantly lower BMI compared to tetraplegics (p 0.002). Lean body mass were decreased for low paraplegics compared to higher. Fat mass was increased for high paraplegics compared to lower.	Study characteristics are similar to Dionyssiotis 2008. This study was deemed to be coming from a similar cohort. The study also focused on paraplegia.
Farkas 2018 [46]	CS (US)	47 (80.1)	43.8 (11.5)	14.4 (11.6)	BMI, subcutaneous adipose tissue and visceral adipose tissue	Visceral adipose tissue was higher in tetraplegia than paraplegia. IL6 was higher in tetraplegia than paraplegia. SBP and total cholesterol was higher in while glucose was lower in paraplegia.	Possible double reporting from another study.
Gorgey 2011B [47]	CS (US)	13 (100)	35.2 (8.6)	12.0 (7.6)	BMI, subcutaneous adipose tissue, visceral adipose tissue	There is no difference in visceral and subcutaneous adipose tissue. BMI is higher for paraplegia (24 ± 4 kg/m2) compared to tetraplegia (21 ± 4.5 kg/m2)	Possible double reporting from another study (Gorgey 2011A)
Groah 2011 [48]	CS (US)	121 (80.2)	37 (12)	11 (8)	BMI	Body mass index was lower in tetraplegics compared to paraplegics	Measured estimates did not report measures of spread (range, interquartile range or standard deviation
Groah 2009 [49]	CS (US)	73 (83.5)	38 (n.a.)	n.a.	BMI	Body mass index was higher in tetraplegia compared to paraplegia	Study characteristics are similar to Groah 2011. This study was deemed to be coming from similar cohort
Gill 2020 [50]	CS (US)	36 (100)	37 (11)	9 (9)	BMI, waist circumference	Higher BMI and weight, but lower waist circumference for paraplegia than tetraplegia. All did not reach statistical significance	Possible double publication from Sumrell 2018.
Hatchett 2016 [51]	CS (US)	222 (89.1)	33.9 (9.7)	9 (9.8)	BMI	No significant difference was observed between groups (paraplegia versus tetraplegia) on baseline and across 3 years	No disaggregated data reported. Secondary analyses of past publication
Janssen 1994 [52]	CS (Netherlands)	43 (100)	11.3 (4.9)	34.4 (5.2)	BMI	No significant difference on body mass among different levels of injury	Study characteristics are similar to Janssen 1997. This study was deemed to be coming from a similar cohort
Liang 2008 [53]	CS (US)	129 (100)	37 (4.0)	n.a.	BMI, waist circumference	No mention on body morphology outcome difference	No disaggregated data for body mass index and waist circumference between tetraplegics and paraplegics
Maruyama 2008 [54]	CS (Japan)	44 (100)	57 (13)	22.3 (10.5)	BMI, waist circumference, fat mass, lean mass	No mention on body morphology outcomes difference	No disaggregated data on fat mass and lean mass between tetraplegics and paraplegics

Sabour 2011[55]	CS (IR)	162 (80.9)	34.17 (n.d.)	n.d.	BMI	Tetraplegia mean BMI 22.66 and paraplegia mean BMI 24.44, higher BMI in paraplegia	Double reporting
Singh 2014 [56]	CH (India)	95(74.7)	33.3 (19	n.a.	BMI, fat mass, lean mass	The tetraplegic patients had significant decrease in arm lean body mass ($p < 0.01$) and fat percentage ($p < 0.01$) compared to paraplegics.	No measurement was given for tetraplegic and paraplegic
Steinberg 2000 [57]	CC (Brazil)	26 (100)	n.a.	n.a.	BMI	No mention on body morphology outcomes difference	Results on body mass index was given per individual

Table S3. Pooled estimates for body composition measured by dual x-ray absorptiometry comparing tetraplegia and paraplegia

Outcome	Number of studies	High injury, N	High injury, mean (SD)	Low injury, N	Low injury, mean (SD)	Weighted mean difference (95% CI)	I2	P value for heterogeneity
Tetraplegia versus paraplegia								
Whole body composition								
Total body fat percentage (%)	7	281	33.9 (7.8)	354	32.6 (8.3)	1.0 (-0.94, 2.96)	61.2%	0.017
Total body fat mass	4	152	26.7 (8.3)	190	24.9 (8.0)	1.2 (-1.6, 4.1)	57.6%	0.069
Total lean body mass (kg)	7	266	46.7 (7.9)	346	48.5 (8.0)	-1.69 (-5.65, 2.27)	87.4%	<0.001
Regional body composition								
Trunk fat percentage	5	171	30.6 (6.7)	226	30.3 (7.2)	-0.32 (-2.92, 2.82)	66.6%	0.017
Trunk fat mass (kg)	4	158	12.8 (4.5)	197	11.5 (3.9)	0.43 (-1.06, 1.92)	60.4%	0.056
Trunk lean mass (kg)	3	93	24.3 (2.2)	116	24.5 (3.2)	-0.51 (-3.11, 2.10)	81.4%	0.005
Leg fat percentage	3	92	36.0 (4.1)	128	38.6 (9.1)	-3.77 (-12.53, 4.98)	90.3%	<0.001
Leg fat mass (kg)	2	73	8.2 (1.0)	92	7.9 (1.8)	-0.03 (-0.04, -0.02)	0.0%	0.390

Table S4. Subgroup analysis based on study characteristics

Study characteristics	Stratum	Number of studies	Standardized mean difference (REML)	I2 for heterogeneity	p value *
Body mass index (kg/m2) TP vs PP					
Individual factors					
Sex	All male	15	-1.448 (-2.083, -0.813)	93.1%	0.030***
	Mixed	15	-0.348 (-1.015, 0.318)	58.7%	
Age	≤ Median	17	-1.336 (-1.918, -0.754)	87.1%	0.082
	> Median	14	-0.456 (-1.226, 0.315)	80.6%	
CV disease/med	Without	14	-1.469 (-2.567, -0.372)	73.3%	0.349
	With CVD	4	-0.050 (-1.065, 0.964)	0.0%	
Injury factors					
Duration of injury	≤ Median	15	-1.303 (-1.873, -0.733)	88.7%	0.194
	> Median	15	-0.665 (-1.676, 0.346)	77.0%	
Completeness of lesion	Mixed	15	-0.375 (-0.913, 0.163)	41.8%	0.201
	100% complete	8	-0.975 (-1.664, -0.285)	72.0%	
Study factors					
Location	Asia	6	-0.961 (-1.748, -0.174)	72.9%	0.226
	Europe	2	-0.250 (-1.791, 1.291)	0.0%	
	North America	20	-0.720 (-1.267, -0.173)	84.6%	
	South America	3	-2.139 (-4.035, -0.243)	79.3%	
Participant	≤ Median	17	-1.669 (-2.556, -0.781)	56.0%	0.026***
	> Median	15	-0.579 (-1.075, -0.084)	90.3%	
Primary outcome	Body composition	14	-0.968 (-1.451, -0.484)	69.6%	0.675
	Other outcomes	17	-0.830 (-1.615, -0.044)	90.0%	
Body mass index (kg/m2) HP vs LP					
Individual factors					
Sex	All male	6	0.053 (-1.047, 1.153)	18.3%	0.517
	Mixed	6	-0.479 (-1.515, 0.556)	33.4%	
Age	≤ Median	5	0.092 (-1.395, 1.578)	21.3%	0.562
	> Median	7	-0.374 (-1.225, 0.477)	29.5%	
CV disease/med	Without	4	-0.436 (-2.173, 1.301)	40.2%	-0.815
	With CVD	2	0.238 (-0.977, 1.452)	0.0%	
Injury factors					
Duration of injury	≤ Median	5	-0.635 (-2.405, 1.135)	47.5%	0.430
	> Median	6	0.226 (-0.639, 1.091)	0.0%	
Completeness of lesion	Mixed	5	-0.771 (-2.362, 0.821)	35.7%	0.685
	100% complete	3	-0.173 (-2.217, 1.871)	52.5%	
Study factors					
Location	Asia	4	0.245 (-1.561, 2.051)	36.6%	0.222
	Europe	2	-1.56 (-3.23, 0.11)	0.0%	
	North America	4	-0.457 (-1.291, 0.378)	9.9%	
	South America	2	1.149 (-0.709, 3.007)	0.0%	
Participant	≤ Median	6	-0.809 (-1.814, 0.197)	19.0%	0.086
	> Median	6	0.357 (-0.504, 1.218)	0.0%	
Primary outcome	Body composition	3	-0.158 (-2.131, 1.814)	55.9%	0.954
	Other outcomes	9	-0.254 (-1.041, 0.534)	17.5%	
Body Fat percentage TP vs PP					
Individual factors					
Sex	All male	2	1.527 (-2.286, 5.340)	45.7%	0.984
	Mixed	8	1.565 (-0.416, 3.546)	57.2%	
Age	≤ Median	4	-0.947 (-5.406, 3.512)	77.0%	0.160
	> Median	7	2.597 (1.478, 3.716)	0.0%	
CV disease/med	Without	3	1.709 (-2.249, 5.667)	1.0%	0.614
	With CVD	1	-0.800 (-7.573, 5.973)	-	
Injury factors					
Duration of injury	≤ Median	4	2.649 (2.060, 3.238)	0.0%	0.205
	> Median	7	0.527 (-2.037, 3.091)	58.9%	
Completeness of lesion	Mixed	4	2.567 (1.375, 3.759)	26.4%	0.403
	100% complete	3	0.741 (-2.925, 4.407)	9.3%	
Study factors					
Location	Asia	2	2.400 (0.288, 4.512)	14.4%	0.040***
	Europe	1	-5.100 (-9.724, -0.476)	-	
	North America	8	2.505 (1.759, 3.250)	3.0%	
	South America	1	1.40 (-1.92, 4.72)	-	
Participant	≤ Median	7	0.605 (-2.703, 3.914)	55.3%	0.218
	> Median	4	2.566 (1.999, 3.133)	0.0%	
Primary outcome	Body composition	10	1.724 (0.381, 3.068)	50.9%	0.469
	Other outcomes	1	4.500 (-1.712, 10.712)	-	
Waist circumference TP vs PP					
Individual factors					
Sex	All male	3	2.258 (-3.577, 8.092)	0.0%	0.990
	Mixed	6	2.402 (-0.042, 4.846)	14.5%	
Age	≤ Median	4	-1.286 (-4.398, 1.825)	0.0%	0.012***
	> Median	6	1.000 (-5.704, 7.704)	0.0%	
CV disease/med	Without	5	0.530 (-3.529, 4.590)	39.4%	0.372
	With CVD	2	4.003 (-2.064, 10.069)	0.0%	

Injury factors					
Duration of injury	≤ Median	4	-1.286 (-4.398, 1.825)	0.0%	**0.012*** **
	> Median	6	1.000 (-5.704, 7.704)	0.0%	
Completeness of lesion	Mixed	5	1.694 (-1.985, 5.373)	54.2%	0.958
	100% complete	2	1.420 (-4.269, 7.109)	0.0%	
Study factors					
Location	Asia	2	-2.051 (-6.037, 1.934)	12.4%	**0.010*** **
	Europe	-	-	-	
	North America	8	3.672 (1.193, 6.151)	0.0%	
	South America	-	-	-	
Participant	≤ Median	5	0.478 (-3.366, 4.323)	0.0%	0.419
	> Median	5	2.593 (-0.273, 5.460)	26.5%	
Primary outcome	Body composition	7	1.568 (-0.704, 3.841)	0.0%	0.853
	Other outcomes	3	1.502 (-5.800, 8.804)	62.9%	
Weight TP vs PP					
Individual factors					
Sex	All male	12	-1.331 (-2.944, 0.282)	61.8%	**0.003*** **
	Mixed	7	4.021 (0.952, 7.091)	9.6%	
Age	≤ Median	11	-0.204 (-1.944, 1.535)	18.6%	0.686
	> Median	9	0.205 (-2.807, 3.217)	54.2%	
CV disease/med	Without	9	-1.308 (-4.660, 2.043)	28.8%	0.989
	With CVD	2	-1.147 (-9.384, 7.090)	0.0%	
Injury factors					
Duration of injury	≤ Median	11	-1.303 (-2.968, 0.362)	65.5%	**0.018*** **
	> Median	8	2.745 (-1.189, 6.679)	33.5%	
Completeness of lesion	Mixed	8	1.360 (-0.233, 2.952)	9.0%	**<0.001*** **
	100% complete	6	-1.669 (-2.470, -0.869)	0.0%	
Study factors					
Location	Asia	3	-1.649 (-2.481, -0.816)	0.0%	0.142
	Europe	2	4.963 (-3.953, 13.879)	31.7%	
	North America	11	0.416 (-1.421, 2.252)	26.6%	
	South America	2	-2.739 (-7.459, 1.982)	0.0%	
Participant	≤ Median	11	-1.667 (-4.476, 1.143)	0.0%	0.252
	> Median	9	0.304 (-1.562, 2.170)	74.8%	
Primary outcome	Body composition	10	0.485 (-1.494, 2.465)	34.1%	0.219
	Other outcomes	10	-1.385 (-3.544, 0.774)	17.9%	
Weight HP vs LP					
Individual factors					
Sex	All male	5	1.045 (-2.983, 5.074)	0.0%	0.275
	Mixed	3	-3.111 (-8.577, 2.355)	0.0%	
Age	≤ Median	4	-0.922 (-6.051, 4.206)	0.0%	0.812
	> Median	4	-0.082 (-4.268, 4.104)	0.0%	
CV disease/med	Without	3	-1.425 (-7.465, 4.615)	0.0%	0.767
	With CVD	1	0.980 (-11.519, 13.479)	.	
Injury factors					
Duration of injury	≤ Median	5	-0.781 (-5.500, 3.938)	0.0%	0.646
	> Median	2	0.996 (-4.599, 6.591)	0.0%	
Completeness of lesion	Mixed	4	-1.217 (-6.386, 3.951)	0.0%	0.550
	100% complete	3	1.074 (-3.964, 6.111)	0.0%	
Study factors					
Location	Asia	2	0.224 (-5.783, 6.230)	0.0%	0.800
	Europe	2	0.472 (-8.206, 9.151)	0.0%	
	North America	2	-2.709 (-8.361, 2.943)	0.0%	
	South America	2	1.429 (-5.194, 8.053)	0.0%	
Participant	≤ Median	4	-1.471 (-6.918, 3.977)	0.0%	0.654
	> Median	4	0.160 (-3.876, 4.196)	0.0%	
Primary outcome	Body composition	2	0.697 (-5.943, 7.337)	0.0%	0.719
	Other outcomes	6	-0.767 (-4.483, 2.949)	0.0%	

* Computed by using the study characteristics as a categorical variable to determine statistically significant difference between the subgroup estimates (using meta-regression); TP, tetraplegia; PP, paraplegia

***p value < 0.05

Table S5. Metaregression of individual and injury factors

	Sex (% males)	Age	Complete injury %	Lesion duration
	Beta (95% CI)	Beta (95% CI)	Beta (95% CI)	Beta (95% CI)
Tetraplegia vs paraplegia				
Body mass index (kg/m2)	-0.04 (-0.08, 0.003)	**0.06 (0.004, 0.125)***	-0.01 (-0.03, 0.01)	0.06 (-0.03, 0.16)
Weight (kg)	**-0.21 (-0.34, -0.07)***	0.18 (-0.09, 0.46)	**-0.09 (-0.12, -0.06)***	**0.57 (0.21, 0.92)***
Total body fat percentage (%)	0.02 (-0.18, 0.23)	0.37 (-0.01, 0.75)	-0.07 (-0.18, 0.04)*	-0.28 (-0.68, 0.11)
Waist circumference (cm)	-0.10 (-0.36, 0.16)	**0.31 (0.05, 0.58)***	0.02 (-0.10, 0.15)	**0.65 (0.15, 1.14)***
High paraplegia vs low paraplegia				
Body mass index (kg/m2)	0.04 (-0.02, 0.09)	-0.11 (-0.91, 0.67)	0.01 (-0.05, 0.08)	0.02 (-0.21, 0.18)
Weight (kg)	0.13 (-0.19, 0.44)	-0.12 (-0.91, 0.67)	0.07 (-0.18, 0.31)	-0.17 (-1.44, 1.09)

Table S6. Metaregression of body mass index with other outcomes according to the level of injury

	Tetraplegia		Paraplegia	
	Beta (95% CI)	pvalue	Beta (95% CI)	pvalue
Using BMI as the dependent variable				
BMI with total body fat percent	0.19 (-0.10, 0.47)	0.171	0.23 (0.006, 0.45)	0.045***
BMI with waist circumference	0.15 (0.02, 0.29)	0.032***	0.13 (0.010, 0.26)	0.039***
BMI with weight	0.26 (0.21, 0.31)	0.000***	0.27 (0.22, 0.32)	0.000***
Using total body fat percent as the dependent variable				
Total body fat percent with BMI	1.14 (-0.44, 2.71)	0.136	1.57 (0.18, 2.97)	0.031***

Figure S1. Forrest plot of studies with body composition as outcome

Author, year of publication	No. of estimates	No. of tetraplegic individuals	No. of paraplegic individuals	I^2 for heterogeneity	P value for heterogeneity	WMD (95% CI)
**Screening measurements**						
Body mass index, kg/m^2	15	829	955	86.9%	0.000	−0.80 (−1.40, −0.40)
Weight, kg	11	356	459	57.8%	0.001	0.50 (−1.30, 2.30)
Waist circumference, cm	6	234	229	14.9%	0.310	1.60 (−0.88, 4.10)
**Total body composition**						
Fat mass, kg	6	196	240	48.7%	0.083	1.50 (−0.40, 3.40)
Lean mass, kg	9	253	318	84.1%	0.000	−2.40 (−5.50, 0.60)
Fat percentage	12	729	873	46.7%	0.044	1.70 (0.70, 2.70)
**Regional body composition**						
Trunk fat, %	5	171	226	66.6%	0.017	-0.32 (-2.92, 2.82)
Trunk fat mass, kg	4	158	197	60.4%	0.056	0.43 (−1.06, 1.92)
Trunk lean mass, kg	3	93	116	81.4%	0.005	−0.51 (−3.11, 2.10)
Leg fat, %	3	92	128	90.3%	0.000	−3.77 (−12.53, 4.98)
Leg fat mass, kg	2	73	92	0.0%	0.390	−0.03 (−0.04, −0.02)
**Visceral adiposity**						
Visceral adipose tissue, cm^2	5	96	119	0.0%	0.858	0.24 (0.05, 0.43)
Visceral adipose volume, L	2	20	49	0.0%	0.532	1.05 (0.14, 1.95)
Subcutaneous adipose tissue, cm^2	2	14	21	5.9%	0.303	−0.20 (−0.76, 0.35)
VAT/SAT ratio	2	20	49	0.0%	0.832	−0.18 (−0.03, 0.40)

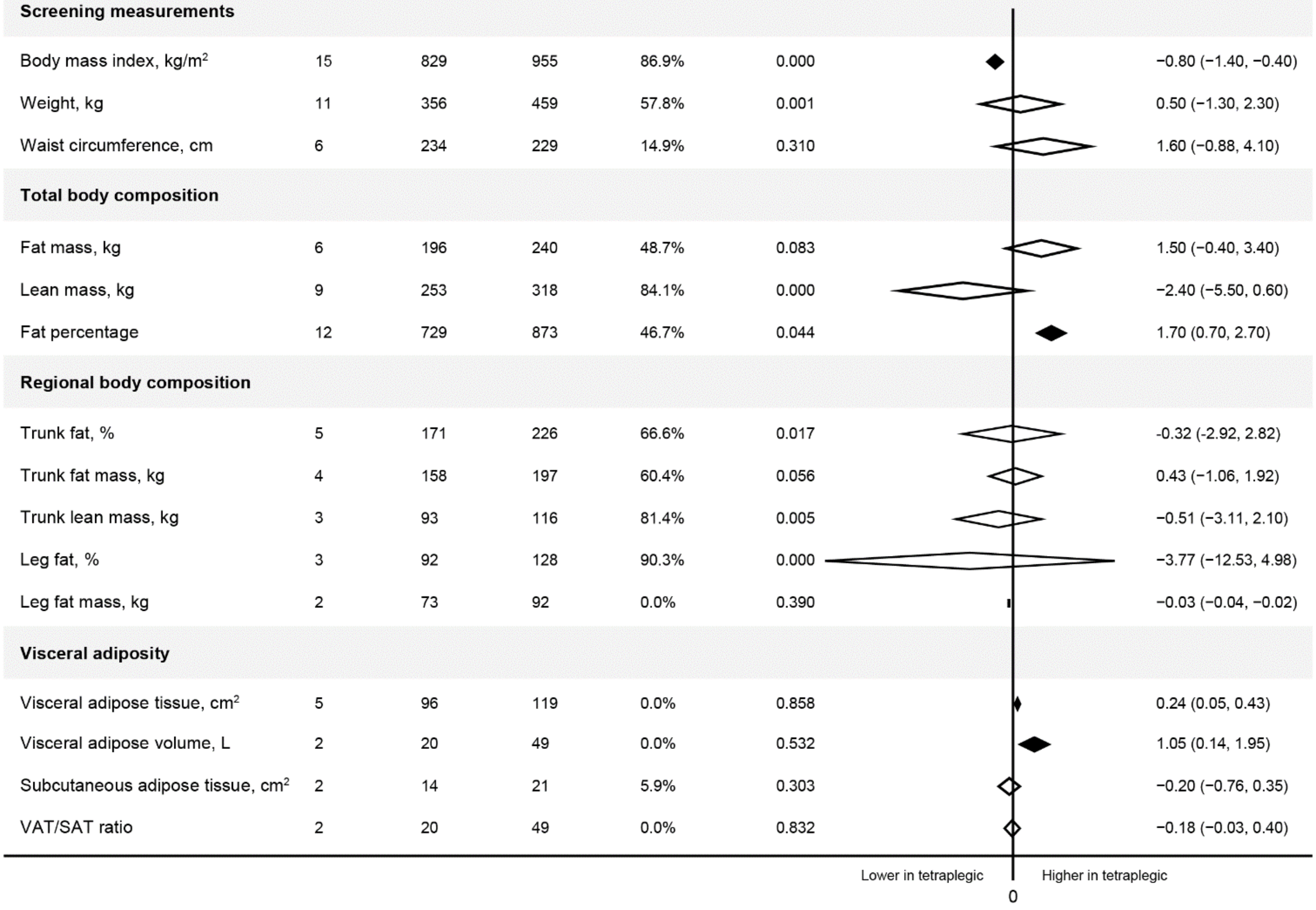

*VAT/SAT (Visceral adipose tissue/Subcutaneous adipose tissue). Black diamond means statistically significant difference (does not cross the line of no-difference) and white diamond means not statistically significant difference (crosses the line of no difference).

When pooling studies in which the body composition/anthropometric measurements were the primary outcome (15 studies for BMI, six studies for waist circumfer-ence, 11 for body weight, 12 studies for total-body fat percentage, six studies for fat mass and nine studies for lean mass), the results were in line with the overall findings of our meta-analysis (Figure 2). Mean total-body fat percentage was higher in individuals with tetraplegia compared to paraplegia (WMD 1.7% 95% CI 0.7, 2.7; 46.7%). Mean body fat mass and was higher in individuals with tetraplegia (WMD 1.5 kg 95% CI -0.4, 3.4; I2 48.7%) lean mass was lower in individuals with paraplegia (WMD -2.4 kg 95% CI -5.5, 0.6; I2 84.1%), but did not reach statistical significance.

Figure S2. Leave-one-out analysis*

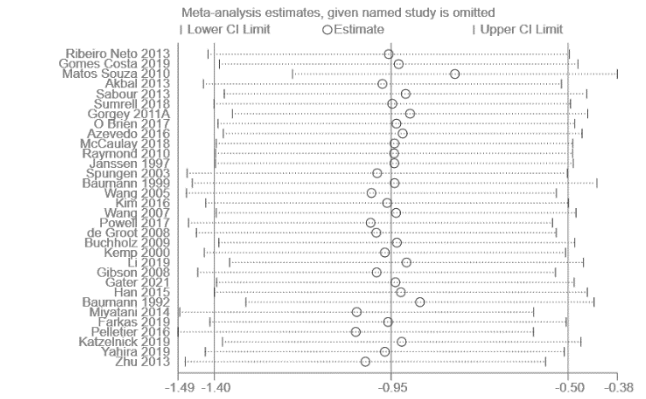

Body mass index (tetraplegia vs paraplegia)

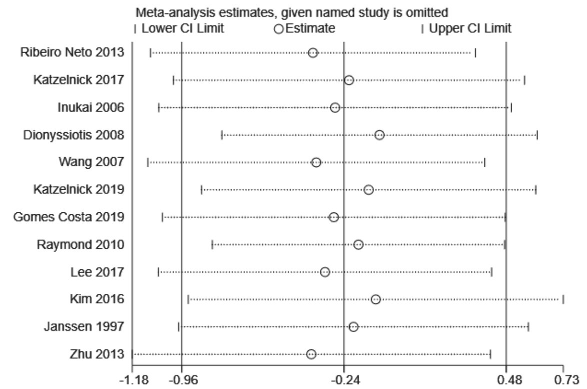

Body mass index (high paraplegia vs low paraplegia)

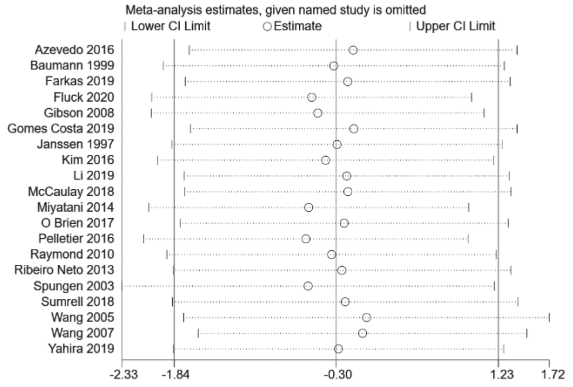

Weight (tetraplegia vs paraplegia)

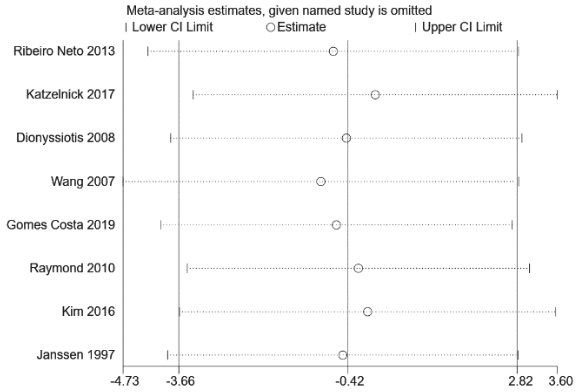

Weight (high paraplegia vs low paraplegia)

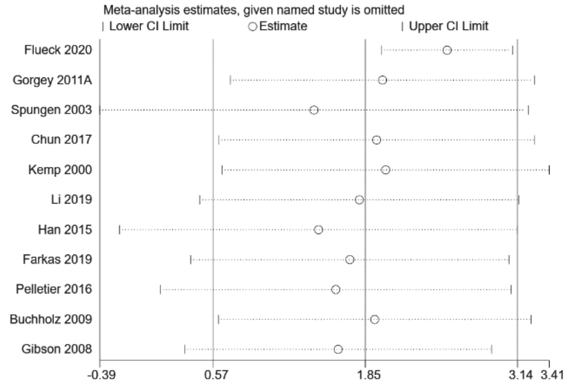

Total fat percentage

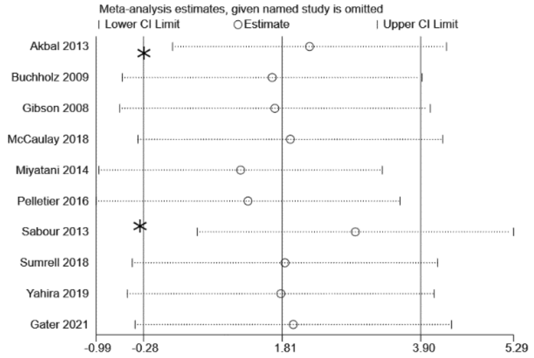

Waist circumference

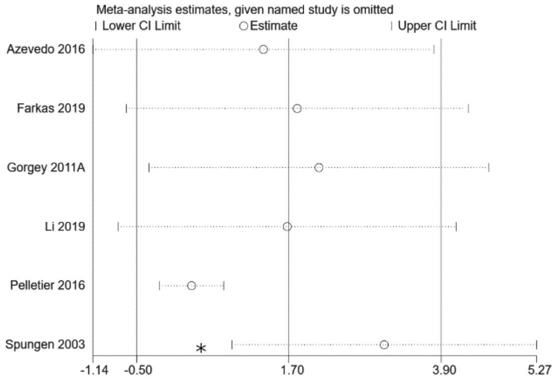

Fat mass

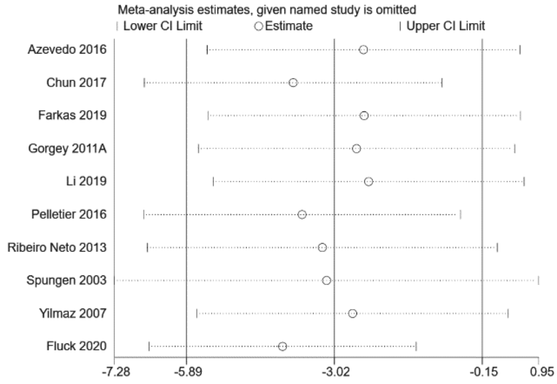

Lean mass

*Leave one out analysis recomputes the weighted mean difference upon removing one study, iteratively. This plot shows the studies removed (y-axis) and the recomputed mean difference/effect estimate (x-axis). Solid vertical lines represent the overall estimate/mean difference and the upper and lower limit of the complete analysis.

Each horizontal line represents the recomputed weighted mean difference upon removing the study indicated (circle as the effect estimate and bars as the confidence interval).

Figure S2. Funnel plot and Egger's test*

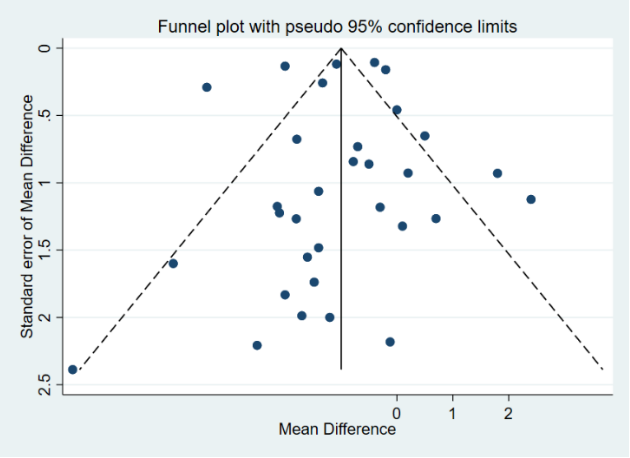

Body mass index (tetraplegia vs paraplegia)
Egger's test 0.897

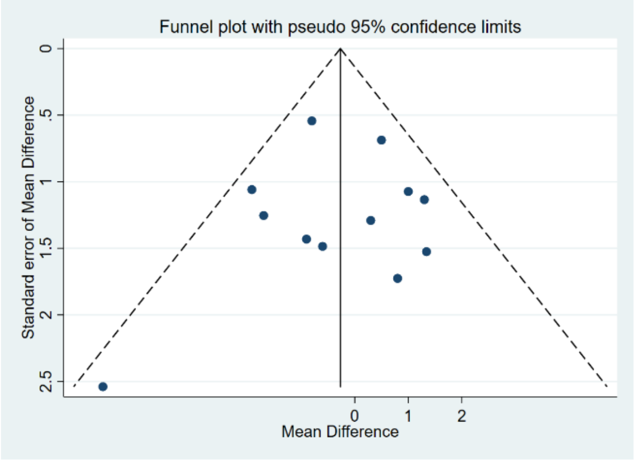

Body mass index (high paraplegia vs low paraplegia)
Egger's test 0.858

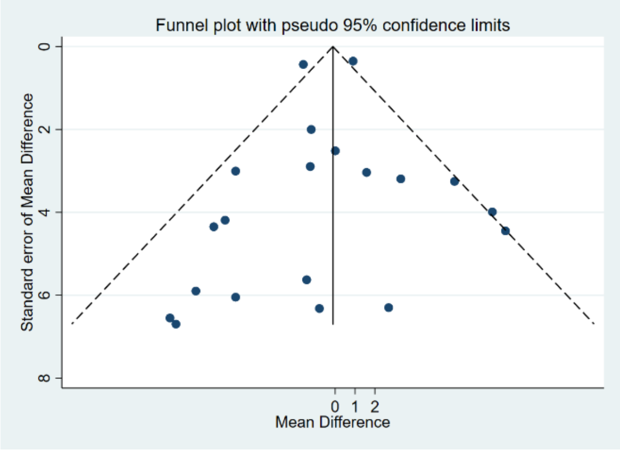

Weight (tetraplegia vs paraplegia)
Egger's test 0.660

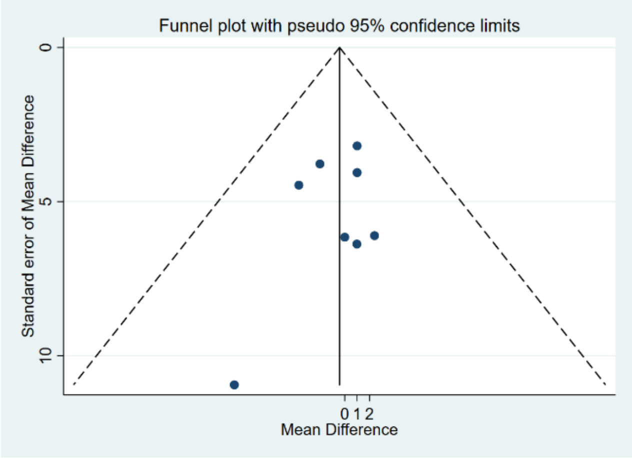

Weight (high paraplegia vs low paraplegia)
Egger's test 0.447

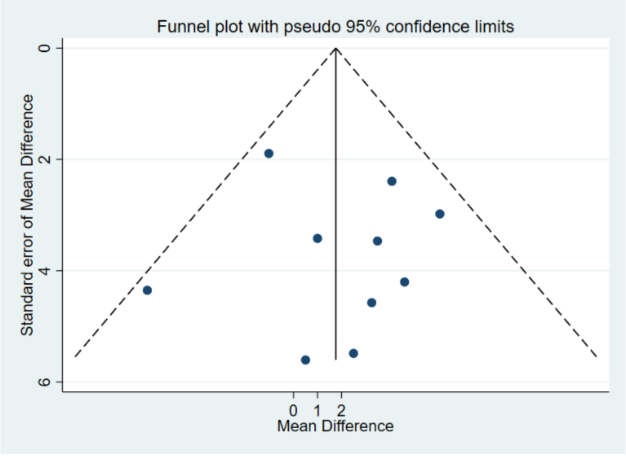

Waist circumference
Egger's test 0.773

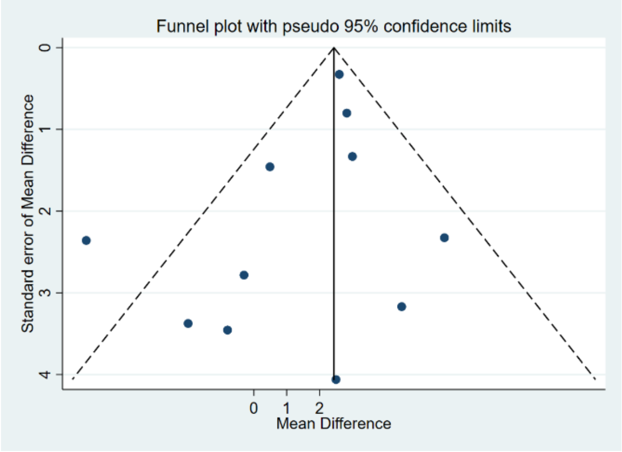

Whole body fat percentage
Egger's test 0.217

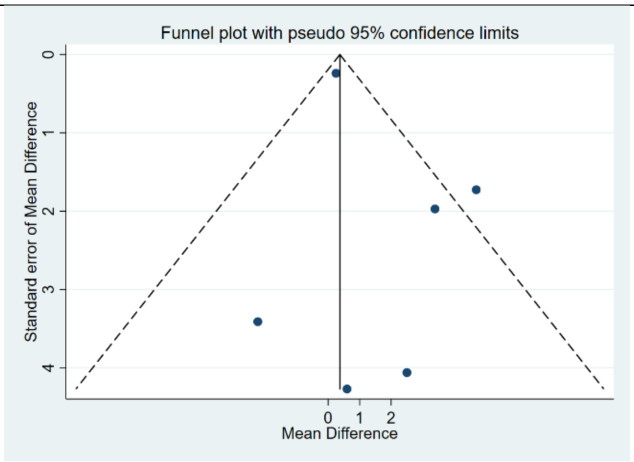

Whole body fat mass
Egger's test 0.279

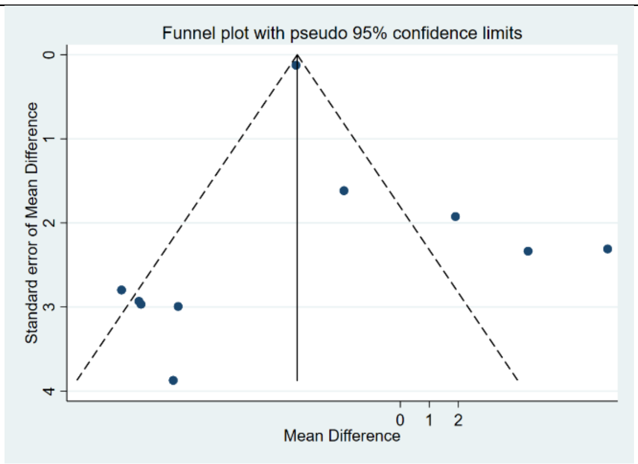

Whole body lean mass
Egger's test 0.860

**Funnel plot visually illustrate the possibility of missing studies (publication bias) by plotting the standard error of mean difference (y axis) and the mean difference (x-axis). The dotted lines illustrate the area in which the studies (or expected studies) lies if there is no heterogeneity and biases.

REFERENCES

1 Akbal A, Kurtaran A, Selcuk B, Akyuz M. H-FABP, cardiovascular risk factors, and functional status in asymptomatic spinal cord injury patients. *Herz*. 2013; 38: 629-35.

2 Azevedo ER, Alonso K, Cliquet A. Body composition assessment by bioelectrical impedance analysis and body mass index in individuals with chronic spinal cord injury. *Journal of Electrical Bioimpediance*. 2016; 7: 2-5.

3 Bauman WA, Adkins RH, Spungen AM, Waters RL. The effect of residual neurological deficit on oral glucose tolerance in persons with chronic spinal cord injury. *Spinal Cord*. 1999; 37: 765-71.

4 Bauman WA, Spungen AM, Zhong YG, Rothstein JL, Petry C, Gordon SK. Depressed serum high density lipoprotein cholesterol levels in veterans with spinal cord injury. *Paraplegia*. 1992; 30: 697-703.

5 Buchholz AC, Martin Ginis KA, Bray SR, Craven BC, Hicks AL, Hayes KC*, et al.* Greater daily leisure time physical activity is associated with lower chronic disease risk in adults with spinal cord injury. *Appl Physiol Nutr Metab*. 2009; 34: 640-7.

6 Chun SM, Kim HR, Shin HI. Estimating the Basal metabolic rate from fat free mass in individuals with motor complete spinal cord injury. *Spinal Cord*. 2017; 55: 844-47.

7 de Groot S, Dallmeijer AJ, Post MW, Angenot EL, van der Woude LH. The longitudinal relationship between lipid profile and physical capacity in persons with a recent spinal cord injury. *Spinal Cord*. 2008; 46: 344-51.

8 Dionyssiotis Y, Petropoulou K, Rapidi CA, Papagelopoulos P, Papaioannou N, Galanos A*, et al.* Body composition in paraplegic men. *J Clin Densitom*. 2008; 11: 437-43.

9 Farkas GJ, Gorgey AS, Dolbow DR, Berg AS, Gater DR. Caloric Intake Relative to Total Daily Energy Expenditure Using a Spinal Cord Injury-Specific Correction Factor: An Analysis by Level of Injury. *Am J Phys Med Rehabil*. 2019; 98: 947-52.

10 Flueck JL. Body Composition in Swiss Elite Wheelchair Athletes. *Front Nutr*. 2020; 7: 1.

11 Gater Jr DR, Farkas GJ, Dolbow DR. Body Composition and Metabolic Assessment After Motor Complete Spinal Cord Injury: Development of a Clinically Relevant Equation to Estimate Body Fat. *Top Spinal Cord Inj Rehabil*. 2021; 27: 11-22.

12 Gibson AE, Buchholz AC, Martin Ginis KA, Group S-SR. C-Reactive protein in adults with chronic spinal cord injury: increased chronic inflammation in tetraplegia vs paraplegia. *Spinal Cord*. 2008; 46: 616-21.

13 Gorgey AS, Gater DR. Regional and relative adiposity patterns in relation to carbohydrate and lipid metabolism in men with spinal cord injury. *Appl Physiol Nutr Metab*. 2011; 36: 107-14.

14 Gomes Costa RR, Carregaro RL, Ribeiro Neto F. Are Body Composition, Strength, and Functional Independence Similarities Between Spinal Cord Injury Classifications? A Discriminant Analysis. *J Sport Rehabil*. 2019: 1-5.

15 Han SH, Lee BS, Choi HS, Kang MS, Kim BR, Han ZA*, et al.* Comparison of Fat Mass Percentage and Body Mass Index in Koreans With Spinal Cord Injury According to the Severity and Duration of Motor Paralysis. *Ann*. 2015; 39: 384-92.

16 Inukai Y, Takahashi K, Wang DH, Kira S. Assessment of total and segmental body composition in spinal cord-injured athletes in Okayama prefecture of Japan. *Acta Med Okayama*. 2006; 60: 99-106.

17 Janssen TW, van Oers CA, van Kamp GJ, TenVoorde BJ, van der Woude LH, Hollander AP. Coronary heart disease risk indicators, aerobic power, and physical activity in men with spinal cord injuries. *Arch Phys Med Rehabil*. 1997; 78: 697-705.

18 Katzelnick CG, Weir JP, Chiaravalloti ND, Wylie GR, Dyson-Hudson TA, Bauman WA*, et al.* Impact of Blood Pressure, Lesion Level, and Physical Activity on Aortic Augmentation Index in Persons with Spinal Cord Injury. *J Neurotrauma*. 2017; 34: 3407-15.

19 Katzelnick CG, Weir JP, Jones A, Galea M, Dyson-Hudson TA, Kirshblum SC*, et al.* Blood Pressure Instability in Persons With SCI: Evidence From a 30-Day Home Monitoring Observation. *Am J Hypertens*. 2019; 32: 938-44.

20 Kemp BJ, Spungen AM, Adkins RH, Krause JS, Bauman WA. The relationships among serum lipid levels, adiposity, and depressive symptomatology in persons aging with spinal cord injury. *J Spinal Cord Med*. 2000; 23: 216-20.

21 Kim JH, Trilk JL, Smith R, Asif I, Maddux PT, Ko YA*, et al.* Cardiac Structure and Function in Elite Para-cyclists with Spinal Cord Injury. *Med Sci Sports Exerc*. 2016; 48: 1431-7.

22 Lee YH, Lee JH, Kim SH, Yi D, Oh KJ, Kim JH*, et al.* Hemodynamic Adaptations to Regular Exercise in People With Spinal Cord Injury. *Ann*. 2017; 41: 25-33.

23 Li J, Hunter GR, Chen Y, McLain A, Smith DL, Yarar-Fisher C. Differences in Glucose Metabolism Among Women With Spinal Cord Injury May Not Be Fully Explained by Variations in Body Composition. *Arch Phys Med Rehabil*. 2019; 100: 1061-67 e1.

24 Matos-Souza JR, Pithon KR, Ozahata TM, Oliveira RT, Teo FH, Blotta MH*, et al.* Subclinical atherosclerosis is related to injury level but not to inflammatory parameters in spinal cord injury subjects. *Spinal Cord*. 2010; 48: 740-4.

25 McCauley LS, Sumrell RM, Gorgey AS. Anthropometric Prediction of Visceral Adipose Tissue in Persons With Motor Complete Spinal Cord Injury. *PM R*. 2018; 10: 817-25 e2.

26 Miyatani M, Szeto M, Moore C, Oh PI, McGillivray CF, Catharine Craven B. Exploring the associations between arterial stiffness and spinal cord impairment: A cross-sectional study. *J Spinal Cord Med*. 2014; 37: 556-64.

27 O'Brien LC, Chen Q, Savas J, Lesnefsky EJ, Gorgey AS. Skeletal muscle mitochondrial mass is linked to lipid and metabolic profile in individuals with spinal cord injury. *Eur J Appl Physiol*. 2017; 117: 2137-47.

28 Pelletier CA, Miyatani M, Giangregorio L, Craven BC. Sarcopenic Obesity in Adults With Spinal Cord Injury: A Cross-Sectional Study. *Arch Phys Med Rehabil*. 2016; 97: 1931-37.

29 Powell D, Affuso O, Chen Y. Weight change after spinal cord injury. *J Spinal Cord Med*. 2017; 40: 130-37.

30 Rankin KC, O'Brien LC, Segal L, Khan MR, Gorgey AS. Liver Adiposity and Metabolic Profile in Individuals with Chronic Spinal Cord Injury. *Biomed Res Int*. 2017; 2017: 1364818.

31 Raymond J, Harmer AR, Temesi J, van Kemenade C. Glucose tolerance and physical activity level in people with spinal cord injury. *Spinal Cord*. 2010; 48: 591-6.

32 Ribeiro Neto F, Lopes GHR. Analysis of body composition values in men with different spinal cord injury levels. *Fisioterapia em Movimento*. 2013; 26: 745-51.

33 Sabour H, Javidan AN, Ranjbarnovin N, Vafa MR, Khazaeipour Z, Ghaderi F*, et al.* Cardiometabolic risk factors in Iranians with spinal cord injury: analysis by injury-related variables. *J Rehabil Res Dev*. 2013; 50: 635-42.

34 Spungen AM, Adkins RH, Stewart CA, Wang J, Pierson RN, Jr., Waters RL*, et al.* Factors influencing body composition in persons with spinal cord injury: a cross-sectional study. *J Appl Physiol (1985)*. 2003; 95: 2398-407.

35 Sumrell RM, Nightingale TE, McCauley LS, Gorgey AS. Anthropometric cutoffs and associations with visceral adiposity and metabolic biomarkers after spinal cord injury. *PLoS One*. 2018; 13: e0203049.

36 Wang YH, Huang TS, Liang HW, Su TC, Chen SY, Wang TD. Fasting serum levels of adiponectin, ghrelin, and leptin in men with spinal cord injury. *Arch Phys Med Rehabil*. 2005; 86: 1964-8.

37 Wang TD, Wang YH, Huang TS, Su TC, Pan SL, Chen SY. Circulating levels of markers of inflammation and endothelial activation are increased in men with chronic spinal cord injury. *J Formos Med Assoc*. 2007; 106: 919-28.
38 Yahiro A, Wingo B, Kunwor S, Parton J, Ellis A. Classification of obesity, cardiometabolic risk, and metabolic syndrome in adults with spinal cord injury. *J Spinal Cord Med*. 2019; 43: 485-96.
39 Yilmaz B, Yasar E, Goktepe AS, Onder ME, Alaca R, Yazicioglu K*, et al.* The relationship between basal metabolic rate and femur bone mineral density in men with traumatic spinal cord injury. *Arch Phys Med Rehabil*. 2007; 88: 758-61.
40 Zhu C, Galea M, Livote E, Signor D, Wecht JM. A retrospective chart review of heart rate and blood pressure abnormalities in veterans with spinal cord injury. *J Spinal Cord Med*. 2013; 36: 463-75.
41 Astorino TA, Harness ET, Witzke KA. Chronic activity-based therapy does not improve body composition, insulin-like growth factor-I, adiponectin, or myostatin in persons with spinal cord injury. *J Spinal Cord Med*. 2015; 38: 615-25.
42 Bauman WA, Spungen AM. Disorders of carbohydrate and lipid metabolism in veterans with paraplegia or quadriplegia: a model of premature aging. *Metabolism*. 1994; 43: 749-56.
43 Bernardi M, Fedullo AL, Di Giacinto B, Squeo MR, Aiello P, Dante D*, et al.* Cardiovascular Risk Factors and Haematological Indexes of Inflammation in Paralympic Athletes with Different Motor Impairments. *Oxid Med Cell Longev*. 2019; 2019: 6798140.
44 Brenes G, Dearwater S, Shapera R, LaPorte RE, Collins E. High density lipoprotein cholesterol concentrations in physically active and sedentary spinal cord injured patients. *Arch Phys Med Rehabil*. 1986; 67: 445-50.
45 Dionyssiotis Y, Lyritis GP, Papaioannou N, Papagelopoulos P, Thomaides T. Influence of neurological level of injury in bones, muscles, and fat in paraplegia. *J Rehabil Res Dev*. 2009; 46: 1037-44.
46 Farkas GJ, Gorgey AS, Dolbow DR, Berg AS, Gater DR. The influence of level of spinal cord injury on adipose tissue and its relationship to inflammatory adipokines and cardiometabolic profiles. *J Spinal Cord Med*. 2018; 41: 407-15.
47 Gorgey AS, Gater DR. A preliminary report on the effects of the level of spinal cord injury on the association between central adiposity and metabolic profile. *PM R*. 2011; 3: 440-6.
48 Groah SL, Nash MS, Ward EA, Libin A, Mendez AJ, Burns P*, et al.* Cardiometabolic risk in community-dwelling persons with chronic spinal cord injury. *J Cardiopulm Rehabil Prev*. 2011; 31: 73-80.
49 Groah SL, Nash MS, Ljungberg IH, Libin A, Hamm LF, Ward E*, et al.* Nutrient intake and body habitus after spinal cord injury: an analysis by sex and level of injury. *J Spinal Cord Med*. 2009; 32: 25-33.
50 Gill S, Sumrell RM, Sima A, Cifu DX, Gorgey AS. Waist circumference cutoff identifying risks of obesity, metabolic syndrome, and cardiovascular disease in men with spinal cord injury. *PLoS One*. 2020; 15: e0236752.
51 Hatchett PE, Mulroy SJ, Eberly VJ, Haubert LL, Requejo PS. Body mass index changes over 3 years and effect of obesity on community mobility for persons with chronic spinal cord injury. *J Spinal Cord Med*. 2016; 39: 421-32.
52 Janssen TW, van Oers CA, van der Woude LH, Hollander AP. Physical strain in daily life of wheelchair users with spinal cord injuries. *Med Sci Sports Exerc*. 1994; 26: 661-70.
53 Liang H, Mojtahedi MC, Chen D, Braunschweig CL. Elevated C-reactive protein associated with decreased high-density lipoprotein cholesterol in men with spinal cord injury. *Arch Phys Med Rehabil*. 2008; 89: 36-41.
54 Maruyama Y, Mizuguchi M, Yaginuma T, Kusaka M, Yoshida H, Yokoyama K*, et al.* Serum leptin, abdominal obesity and the metabolic syndrome in individuals with chronic spinal cord injury. *Spinal Cord*. 2008; 46: 494-9.
55 Sabour H, Javidan AN, Vafa MR, Shidfar F, Nazari M, Saberi H*, et al.* Obesity predictors in people with chronic spinal cord injury: an analysis by injury related variables. *J Res Med Sci*. 2011; 16: 335-9.
56 Singh R, Rohilla RK, Saini G, Kaur K. Longitudinal study of body composition in spinal cord injury patients. *Indian j*. 2014; 48: 168-77.
57 Steinberg LL, Lauro FA, Sposito MM, Tufik S, Mello MT, Naffah-Mazzacoratti MG*, et al.* Catecholamine response to exercise in individuals with different levels of paraplegia. *Braz J Med Biol Res*. 2000; 33: 913-8.
